# Substance use in people at clinical high-risk for psychosis

**DOI:** 10.1186/s12888-014-0361-1

**Published:** 2014-12-24

**Authors:** Debra A Russo, Jan Stochl, Michelle Painter, Peter B Jones, Jesus Perez

**Affiliations:** CAMEO Early Intervention in Psychosis Service, Cambridgeshire and Peterborough NHS Foundation Trust, Cambridge, UK; Department of Psychiatry, University of Cambridge, Cambridge, UK; Department of Health Sciences, University of York, York, UK; NIHR Collaboration for Leadership in Applied Health Research & Care, Cambridge, UK; Block 7, Ida Darwin Site, Fulbourn Hospital, CB21 5EE Fulbourn, Cambridge UK

**Keywords:** Alcohol, Cannabis, High-risk, Psychosis, Substance use

## Abstract

**Background:**

Some high-risk (HR) mental states for psychosis may lack diagnostic specificity and predictive value. Furthermore, psychotic-like experiences found in young populations may act not only as markers for psychosis but also for other non-psychotic psychiatric disorders. A neglected consideration in these populations is the effect of substance misuse and its role in the development of such mental states or its influence in the evolution toward full psychotic presentations. Therefore, the main aim of this study was to thoroughly describe past and current substance use profiles of HR individuals by comparing a consecutive cohort of young people at high risk referred to a population-based early intervention clinical service with a random sample of healthy volunteers (HV) recruited from the same geographical area.

**Methods:**

We compared alcohol and substance use profiles of sixty help-seeking HR individuals and 60 healthy volunteers (HV). In addition to identification of abuse/dependence and influence on psychotic-like experiences, differences between HR individuals and HV were assessed for gender, ethnicity, occupational status, age of lifetime first substance use, prevalence and frequency of substance use.

**Results:**

There were no cases of substance use disorder or dependence in either groups. HR individuals were significantly younger than HV when they first started to use substances (p = 0.014). The prevalence of overall HR substance use was similar to that of HV. Although HR individuals reported less cannabinoid use than HV currently (15% vs. 27%), and more in the past (40% vs. 30%), the differences were not statistically significant (p = 0.177 & 0.339 respectively). Current frequency of use was significantly higher for HR individuals than HV for alcohol (p = 0.001) and cannabinoids (p = 0.03). In this sample, only 5% of HR individuals converted to psychosis over a two-year follow-up.

**Conclusions:**

Certain profiles of substance use could potentially play a significant part in the evolution of HR presentations. Therefore, substance use may well represent a clinical domain that requires further emphasis and more detailed consideration in future studies.

## Background

It is noteworthy that overall transition rates reported in different cohorts of individuals at clinical high-risk for psychosis (HR) have consistently declined over the last decade [1]. Also, conversion rates have varied across different centers world-wide [1,2]. These discrepancies have been associated with a variety of factors. For example, it has been suggested that the ultimate level of current conversions may not be so low or diverse if high risk individuals were monitored for both longer and comparable follow-up periods [2]. In addition, early detection might indirectly involve provision of non-specific clinical care. Supportive therapy and/or pharmacological interventions, including antidepressants or anxiolytics could reduce stress and subsequently, the likelihood of conversion into frank psychotic disorders. Also, by detecting this group earlier some recent cohorts may have included more false positives than previous studies. In other words, early detection of these mental states may also identify HR phenotypes that could eventually take different diagnostic trajectories [1,2]. Accordingly, some HR mental states for psychosis may lack diagnostic specificity and predictive value. In fact, presence of psychotic-like symptoms in young people with disorders of anxiety and depression is more prevalent than previously considered [3,4]. Furthermore, psychotic-like experiences found in adolescent populations may act not only as markers for psychosis but also for other non-psychotic psychiatric disorders [5].

Notably, none of these hypotheses have considered the effect of substance misuse in HR individuals and its role in the development of such mental states or its influence in the evolution toward full psychotic presentations. This seems particularly pertinent as alcohol and drug misuse is common among people with psychotic illnesses, including those suffering from a first-episode, and significantly more prevalent than in the general population [6-8]. Moreover, the abuse of illicit substances, such as cannabis, has been positively associated with the development of psychotic disorders [9,10]. A recent literature review suggested that increased rates of substance misuse in HR individuals may be associated with transitions to psychosis. However, it was also highlighted that this evidence was limited by the low number of studies that considered this variable, variety of results and scarce information regarding change of patterns of use over time. Moreover, the vast majority of studies evaluated in this review neither recorded alcohol misuse nor included a comparative group of representative healthy volunteers (HV) in order to better determine possible differences with regard to substance use habits in those individuals at HR [11].

This review also revealed that only diagnostic structured interviews were employed to assess substance use. These tools exclusively focus on the identification of substance abuse and/or dependence [11]. Therefore, it would be preferable to employ a tool to accurately measure alcohol and drug use and enable a complete evaluation of substance use that does not necessarily reach the category of dependence and/or abuse.

Given the paucity of studies primarily addressing the impact of alcohol and drug misuse in HR populations, the main aim of this study was to thoroughly describe past and current substance use profiles of HR individuals by comparing a consecutive cohort of young people at HR referred to a population-based early intervention clinical service with a random sample of HV recruited from the same geographical area.

## Methods

### Setting

CAMEO (http://www.cameo.nhs.uk) is an early intervention in psychosis service which offers management for people aged 14-35 years suffering from first-episode psychosis in Cambridgeshire, UK. CAMEO also accepts referrals of people at HR. Referrals are accepted from multiple sources including general practitioners, other mental health services, school and college counselors, relatives and self-referrals [12].

### Sample

A consecutive cohort of 60 help-seeking individuals, aged 16-35, referred to CAMEO from February 2010 to September 2012 met criteria for HR, according to the Comprehensive Assessment of At Risk Mental States (CAARMS) [13]. In our sample, all individuals fulfilled criteria for the attenuated psychotic symptoms group. Seven individuals (11.7%) also qualified for the vulnerability traits group. The only exclusion criteria were confirmed intellectual disability (Wechsler Adult Intelligence Scale – tested IQ <70), or prior total treatment with antipsychotics for more than one week.

During the same period (February 2010-September 2012), a random sample of 60 HV was recruited by post, using the Postal Address File (PAF®) provided by Royal Mail, UK. To ensure that each HR and HV resided in the same geographical location, 50 corresponding postcodes, matching the first 4/5 characters and digits of each recruited HR individual (e.g. PE13 5; CB5 3), were randomly selected using Microsoft SQL Server, a relational database management system, in conjunction with the PAF database. Each of these 50 addresses was sent a recruitment flyer containing a brief outline of the study, inclusion criteria and contact details. If this failed to generate recruits, a consecutive sample of postcodes was selected. This process was repeated until a match was recruited. HV interested in the study could only participate if they were aged 16-35, resided in the same geographical area as HR individuals (Cambridgeshire), and did not have previous contact with mental health services.

### Ethical approval

Ethical approval was granted by the Cambridgeshire East Research Ethics Committee.

### Measures

Sociodemographic information (age, gender, ethnicity and occupational status) was collected for all individuals.

HR individuals were interviewed by senior trained psychiatrists working in CAMEO, using the Mini International Neuropsychiatric Interview (MINI), Version 6.0.0 [14], a brief structured diagnostic interview for DSM-IV Axis I psychiatric disorders.

The Positive and Negative Syndrome Scale (PANSS) [15] for psychotic symptoms was also employed to capture the severity of positive symptoms (7 items), negative symptoms (7 items) and general psychopathology (16 items) in a 7-point scale, with higher scores indicating greater severity of illness. These assessments were carried out by senior research clinicians trained to administer each of the measurement tools.

A novel substance use tool was used to record the specific type of drug and categorised it according to chemical constituents; these comprised sedatives, hallucinogens, dissociatives, cannabinoids, stimulants, opiates, solvents, alcohol and other substances (e.g. legal highs). Frequency was measured using 8 categories: never, one off, less than once a month, once a month, once or twice a week, 3-6 times a week, daily use and uncertain frequency. Quantity measures were excluded as they could lack validity due to the possible inaccuracy in self-reports of drug purity, variety and the size of drug doses. Age at first use was also recorded as age of first substance use has been found to predate initial psychotic symptoms by several years [8,10] and has been associated with the onset of prodromal symptoms [10,16]. It has been suggested that individuals may use substances to self-medicate following the onset of psychotic symptoms [17]. Conversely, it has been argued that substance misuse might cause psychotic symptoms or increase the likelihood of psychotic symptoms in already vulnerable individuals [10,18,19]. Therefore, questions were added to capture a) whether any unusual experiences were experienced under the influence of drugs or alcohol and b) whether drugs or alcohol were used to relieve any unusual symptoms. Individuals were asked about their current drug and alcohol use (now and within the last 3 months) and their greatest past use (period of time prior to the last three months when drug and alcohol use was at its greatest). It was not possible to discern the extent to which individuals deny or exaggerate alcohol and drug use. To minimise this, participants were assessed during a face to face interview which took place over several sessions. This provided confidentiality and enabled the interviewer to build a rapport with the participant, both of which have been shown to increase the validity of self-report [20].

### Statistical analysis

Differences between HR individuals and HV were assessed using two sample *t*-test for approximately normally distributed continuous variables (age) and Fisher’s exact test for categorical variables (gender, ethnicity and occupational status). Fisher’s exact test was also used for assessing the differences between substance use distributions and patterns as this is more appropriate for smaller sample sizes. Wilcoxon signed rank test was employed for non-normally distributed continuous variables (age of lifetime first substance use, frequency of substance use). Boxplots were used for graphical representation of the differences in frequency of substance use.

## Results

### Sociodemographic profile

Sociodemographic information was collected, comprising age, gender, ethnicity and occupational status. Table 1 shows a comparison between HR and HV individuals. There was a difference in age between the two groups; HV were significantly older than the HR individuals (22.6 SD = 5.7 vs. 19.9 SD = 2.4; p = < 0.001). The HR group had a slightly higher proportion of males and the HV group had a slightly higher proportion of females. Both groups were predominantly white with a similar proportion of Mixed, Asian and Black participants. Both groups contained the same number of students (41.7%), but significantly more HV were employed (p = 0.001).

**Table 1:** **Sociodemographic comparison between HR and HV individuals**

**Sociodemographic characteristics**	**HR (n = 60)**	**HV (n = 60)**	**p-values**
**Age at study entry, years (median, min, max, SD)**	19.89 (16.41, 30.21, 2.38)	22.60 (16.18, 35.57, 5.68)	< 0.001*
**Gender (n, %)**			
**Male**	31 (51.7%)	26 (43.3%)	0.465^~^
**Female**	29 (48.3%)	34 (56.7%)	0.465^~^
**Ethnicity (n, %)†**			
**White**	56 (93.3%)	55 (91.7%)	1.000^~^
**Mixed**	2 (3.3%)	2 (3.3%)	1.000^~^
**Asian**	1 (1.7%)	2 (3.3%)	1.000^~^
**Black**	1(1.7%)	1(1.7%)	1.000^~^
**Occupational status (n, %) (7)‡**			
**Unemployed**	20 (33.3%)	8 (13.%)	0.004^~^
**Employed**	8 (13.3%)	27 (45.0%)	0.001^~^
**Students**	25 (41.7)	25 (41.7)	0.575^~^

‘P- values’ * = *t*-test ^~^ = Fisher’s exact.

† ‘White ethnicity’ refers to subjects who are White British, White Irish, or other White backgrounds.

‘Mixed ethnicity’ refers to those who are White and Black Caribbean, mixed White and Black African, mixed White and Asian, or any other mixed backgrounds.

‘Asian ethnicity’refers to those who are Indian or Chinese.

‘Black ethnicity’ refers to subject from any Black backgrounds.

‡ Occupational status is broadly categorized into 3 groups.

‘Unemployed’ includes subjects who do not have a job, either they are looking for work, not looking for work (e.g., housewife), or not being able to work due to medical reasons.

‘Employed’refers to people who have full/part-time employment, or employed but currently unable to work.

‘Students’ refers to full/part-time students, including those who are also working some hours.

### Psychiatric diagnoses and PANSS scores

We obtained MINI DSM-IV diagnoses for 55 of the 60 HR individuals. Thirty Eight (69.1%) had more than one DSM-IV psychiatric diagnosis, mainly within the affective and anxiety diagnostic spectra. Primary diagnoses for this group were ranked as follows: major depressive episode, current or recurrent (n = 26; 47.3%) > social phobia (n = 7; 12.7%) = generalised anxiety disorder (n = 7; 12.7%) > obsessive compulsive disorder (n = 5; 9.1%) > bipolar disorder, type II (n = 2; 3.6%) > panic disorder (n = 1; 1.8%) = posttraumatic stress disorder (n = 1; 1.8%). Six HR individuals (10.9%) did not fulfill sufficient criteria for a DSM-IV Axis I diagnosis. None of the participants had a substance use disorder. The study protocol did not routinely administer a MINI for HV. However, if the information elicited with the substance use questionnaire indicated that substance use was approaching the threshold for abuse or dependence the protocol was to administer a MINI for verification. This was not the case for any of the HV.

The mean PANSS scores for the HR group comprised positive symptoms (13.1, SD = 3.2), negative symptoms (12.4, SD = 5.0) and general psychopathology (32.7, SD = 7.0). These scores indicated a “mildly ill” group with regards to psychotic symptoms [21]. Psychotic symptoms for the HV group were subclinical: 7.1 (SD = 0.4) for positive symptoms, 7.8 (SD = 0.8) for negative symptoms and 16.4 (SD = 1.3) for general psychopathology.

### Substance use

#### Distribution of substance use

Table 2 shows the number and percentages of individuals who were using each of the substances at the time of their referral to CAMEO. Alcohol and cannabinoids were the most prevalent for both the HR and HV groups.

**Table 2:** **Substance use distribution in HV and HR individuals at the time of referral to CAMEO**

	**HR(n)**	**%**	**HV(n)**	**%**	p-value*
**Alcohol**	18	30.0	31	51.6	0.025
**Cannabinoids**	9	15.0	16	26.6	0.177
**Dissociatives**	1	1.6	0	-	1
**Hallucinogens**	3	5	4	6.6	1
**Opiates**	1	1.6	0	-	1
**Sedatives**	1	1.6	0	-	1
**Stimulants**	6	6	4	6.6	0.743

P- values: * = Fisher’s exact.

Table 3 shows how many of the HR and HV individuals were not using any substances, using only one substance (mono-drug) and more than one substance (poly-drug) currently and in the past. Interestingly, more HR individuals (52%) than HV (12%) indicated that they did not use any substance currently (p = 0.001). Although 42% of HR individuals and 32% of HV abstained from using any substance in the past, this difference was not statistically significant (p = 0.343). A significantly higher proportion of HV disclosed that they were currently using one substance (58% vs. 32%, p = 0.006) but not poly substances (30% vs. 17%, p = 0.131). Similarly, more HV individuals reported using only one substance in the past (p = 0.028). However, the percentage of past poly-drug users was higher for HR individuals (38% vs. 28%), although statistical significance was not reached (p = 0.333).

**Table 3:** **Substance use pattern in HR and HV individuals**

**Current**						**Past**				
	**HR(n)**	**%**	**HV(n)**	**%**	p value*	**HR(n)**	**%**	**HV(n)**	**%**	p-value*
**No**	31	52	7	12	<0.001	25	42	19	32	0.343
**Mono-drug**	19	32	35	58	0.006	12	20	24	40	0.028
**Poly-drug**	10	17	18	30	0.131	23	38	17	28	0.333

P- values: * = Fisher’s exact.

#### Age of lifetime first substance use

When considering all substances, the median age of HR individuals was 13 (SD = 2.2) and 15 (SD = 3.7) for HV. Results of a Wilcoxon signed-rank test revealed that HR individuals were significantly younger than HV when they first started to use substances (p = 0.014). When excluding alcohol, the finding was in the same direction (14, SD = 1.58 vs.16, SD = 2.7; p = 0.020). This suggests that for both groups, initial alcohol consumption happened 1-2 years before drug use commenced.

#### Current prevalence of substance use

Alcohol and cannabinoids were the most prevalent choice of substance for mono-drug and poly-drug users for both groups. Of the 19 HR individuals that reported currently using only one substance 95% used just alcohol and 5% used just cannabinoids. However, 100% of the 13 HV current mono-drug users reported using only alcohol. Table 4 outlines how many of the 10 HR and 18 HV current poly-drug users endorsed the use of each category of substance. Alcohol, cannabinoids and stimulants were the most likely substances of choice for HR poly-drug users; for HV, it was alcohol and cannabinoids. These findings suggest that HR poly-drug users experimented with a wider range of substances than HV poly-drug users.

**Table 4:** **Number of HR and HV individuals that endorsed using each substance for current and past mono-drug and poly-drug use**

	**Current**						**Past**					
	**Mono-drug Users**			**Poly-drug Users**			**Mono-drug Users**			**Poly-drug Users**		
	**HR (n=19)**	**HV (n=35)**	p-value*	**HR (n=10)**	**HV (n=18)**	p-value*	**HR (n=12)**	**HV (n=24)**	p-value*	**HR (n=23)**	**HV (n=17)**	p-value*
**Alcohol**	18	13	0.404	10	18	0.130	8	21	0.010	22	17	0.436
**Cannabinoids**	1	0	1	8	16	0.109	2	2	1	22	16	0.327
**Dissociatives**	0	0	1	1	0	1	0	0	1	6	2	0.272
**Hallucinogens**	0	0	1	3	4	1	1	1	1	6	4	0.743
**Opiates**	0	0	1	1	0	1	0	1	1	3	3	1
**Sedatives**	0	0	1	1	0	1	0	0	1	1	0	1
**Stimulants**	0	0	1	6	4	0.743	1	0	1	15	9	0.254

P- values: * = Fisher’s exact.

#### Past prevalence of substance use

For both HR and HV individuals, there was a wider range of substances used in the past. A higher proportion of HV (40%) reported past mono use of substances when compared with HR mono-drug users (20%, p = 0.028). In addition to alcohol and cannabinoids, HR mono-drug users also experimented with hallucinogens and stimulants and HV mono-drug users with cannabinoids and opiates.

For past poly use of substances, the number of HR individuals reporting use for each substance was higher with the exception of opiates, which was the same. However, none of the differences reached statistical significance (see table 4). There was also an increase in the range of substances for poly-drug use. Hallucinogens, dissociatives and stimulants were additions for HV compared to dissociatives, sedatives and opiates for HR individuals.

When combining mono-drug and poly-drug users, current alcohol use was similar with 47% of HR individuals and 52% of HV endorsing use (p = 0.715). Similarly, there was no significant difference in the amount of alcohol use disclosed by HV (65%) and HR individuals (48%, p = 0.197). For cannabinoids, there were slight differences in current and past use. Fewer HR individuals acknowledged cannabinoid use than HV at the time of their referral to CAMEO (15% vs. 27%), but more HR individuals endorsed use in the past (40% vs. 30%). However, these differences were not statistically significant (p = 0.177 & 0.339 respectively).

#### Frequency of substance use

**Figure 1:**
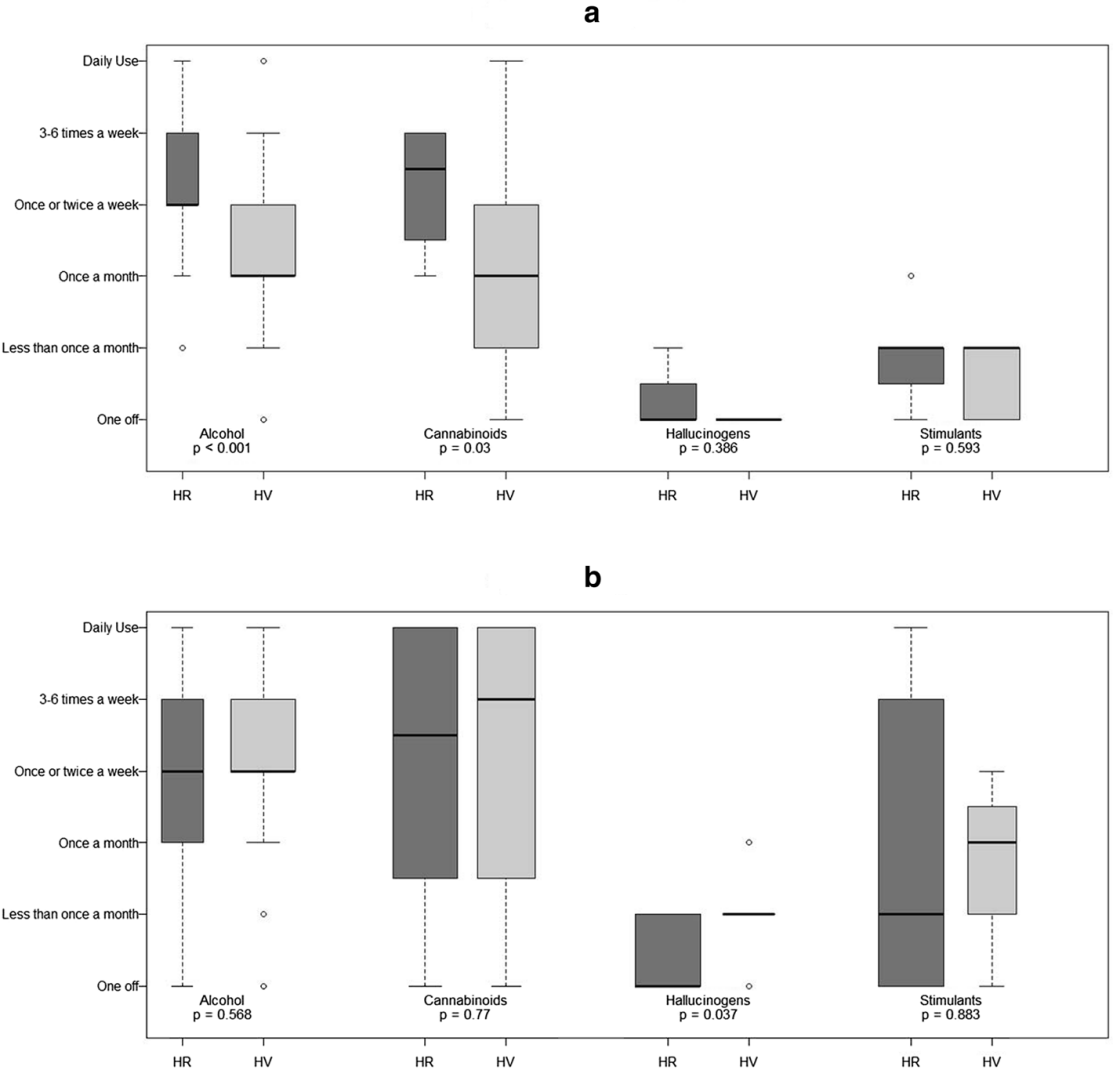
**Frequency of substance use in HR and HV individuals. (a)** Current frequency of substance use **(b)** Past frequency of substance use.

Figure (1a) shows the frequency of current use for the most prominent substances. The median frequency of use was significantly higher for HR individuals than HV for alcohol (p = 0.001) and cannabinoids (p = 0.03), but not for hallucinogens (p = 0.386) and stimulants (p = 0.593). Combined with the previous results, this indicates that although the proportion of HV that drank alcohol and use cannabinoids was higher in general, HR individuals used these substances more frequently.

Figure (1b) shows the frequency of past use for the most prominent substances. There were no significant differences in past frequency of use for any of the substances with the exception of hallucinogens. HV used hallucinogens significantly more often than HR individuals (p = 0.037). This suggests that frequency of substance use for HR individuals remained similar for current and past use; whereas HV were more likely to have a period in the past where they used hallucinogens more frequently.

#### Experience or relief of psychotic-like experiences

Eleven percent of HR individuals reported experiencing psychotic-like symptoms under the influence of substances and 10% reported using substances to help relieve these experiences. All the HV denied psychotic-like experiences under the influence of substances or using substances to help relieve these symptoms.

## Discussion

The main aim of this study was to thoroughly describe past and current substance use profiles of HR individuals and compare them with a sample of healthy volunteers. Results showed that, for overall substance use, the prevalence of HR substance use was less or similar to that of HV. The ony exception to this was past poly-drug use, which was sightly higher for HR individuals, although not statistically significant. HR poly-drug users experimented with a wider range of substances than HV poly-drug users. HR individuals were significantly younger than HV when they started using alcohol and drugs. Choice of substance was similar when comparing HR and HV individuals’ current and past use. Alcohol was the most frequently reported substance used in both groups. In terms of illicit substances, cannabis was the most widely used drug in both groups. The use of other illicit substances was considerably lower compared with cannabis. The least used substances for both groups were sedatives and opiates.

Addington et al.'s recent review of HR individuals revealed that cannabis was the most commonly used substance [11], whereas in the present study it was alcohol. Rates of use varied from 33% to 54%; this was considerably higher than the 9% reporting cannabis use in the present study. However, the prevalence of alcohol use (46.5%) was greater than the highest reported rate in other studies (17% - 44%).

Interestingly, none of the HR or HV individuals included in this study could be categorised as suffering from DSM-IV substance use disorder or dependence. This is not only significantly different to the severity of use reported in other HR samples [11], but also to a population-based sample of individuals experiencing first-episode psychosis from the same early intervention service [8]. In this cross sectional analysis cannabis abuse or dependence and alcohol abuse or dependence was reported in approximately 50% of CAMEO first episode psychosis (FEP) patients. In addition, 38% disclosed poly substance abuse and more than half of them used Class A drugs. These findings were also replicated in FEP samples from other countries [22].

Therefore, the HR substance use profile in the present sample was not only different to HV from the same geographical area, it also appears to differ from first-episode psychosis patients in our region at the time of their referral to CAMEO. This is further substantiated by the fact that after approximately 2 years of an antipsychotic-free follow-up period for each individual at HR in this sample, only 3 (5%) made a transition to a psychotic disorder. One possible conclusion to be drawn is that their pattern of use could have some influence on psychotic-like experiences but not on transition to a frank psychotic disorder. Nevertheless, the frequent diagnosis of mood or anxiety disorders in this sample supplicates the consideration that substance use may also have had an impact these outcomes. However, the cross-sectional design of our study did not allow the consideration of the role substance use in the evolution of other non-psychotic psychiatric disorders.

The main difference between HR individuals and HV was frequency of substance use. Current frequency of use was significantly higher in HR individuals than HV for alcohol and cannabinoids. However, daily use of cannabis in our HR group (0%) was much lower than in other studies, which found this frequency in around 60% of their HR samples [23,24]. Cannabis use once to twice a week occurred in 7% of our HR individuals in comparison to 20% [23] and 19% [24] in previous studies. The one study that reported frequency of alcohol use found similar drinking behaviours in HR and HV individuals [25].

Notably, the frequency of substance use for HR individuals, particularly for alcohol and cannabinoids, remained similar for current and past use; whereas HV were more likely to have a period in the past where they used these substances more frequently. This could suggest that sustained substance use over a protracted period could be more deleterious than a shorter period of increased use. Furthermore, the higher frequency of substance use in HR individuals combined with a significantly younger age of first use might eventually contribute to the development of psychotic-like experiences.

The hypothesis that some individuals may use substances to alleviate psychotic symptoms [17] was not supported in this study. In fact, very few HR individuals reported using substances to help relieve these experiences.

The results of this study must be considered in the light of the following limitations. The multiple incidences of depression and anxiety combined with the lack of transitions may call in to question the authenticity of our HR sample. However, co-morbidity of disorders of anxiety and depression with psychotic symptoms appears to be more prevalent than previously considered in adolescents and young adults [3]. Added to this, the short follow-up in this study could explain the low transition rate. Transitions can occur up to 10 years after psychotic symptoms first emerge [26]. Moreover, the 3 monthly follow-ups in this study may have been therapeutic, indirectly providing non-specific clinical care and consequently reducing the likelihood of transition. Certainly, scrutiny of the follow-up intervals in Addington’s review [11] revealed diverse monitoring periods, in addition to varied transition rates. Therefore, drawing valid conclusions on this issue is complex. Also, the pattern of substance use was not closely monitored for each individual after the time of their referral to CAMEO. Future research should address this limitation since prospective follow-up could reveal changes in patterns of substance use that could have an impact on the incidence of psychotic experiences over time. The small sample size of 60 participants is acknowledged. However, this number is greater or comparable to over half the studies in Addington’s review [11].

The sociodemographic differences in our sample compared to other HR samples in the literature are also potential limitations. Firstly, HV were significantly older than HR individuals. However, the influence of this dissimilarity in the domains that were significantly different between both groups, i.e. age of first substance use and frequency of substance use, was arguably negligible. Secondly, there is a geographical difference compared to other research describing substance use in HR samples. Although the majority of studies in Addington’s review [11] were conducted in USA and Australia, several were conducted in Europe. However, none were exclusively in the UK. Despite the limitations of comparing such a diverse geographical spread of HR samples, describing substance use in a UK sample of HR individuals provides a useful contribution to the literature. Thirdly, although there was some representation of different ethnicities, the sample was predominantly white. Comparisons with the existing literature on substance use in HR samples are problematic as the majority of studies did not report ethnicity or they dichotomised the categories e.g. white vs non-white (see Addington et al. [11]). Finally, while the gender ratio did not differ significantly between HR and HV groups, the slightly higher proportion of males in the HR group may have influenced the patterns of substance use, as male gender is associated with substance use in patients and psychotic disorders in the general population [27].

## Conclusions

Research on individuals at HR is showing a remarkable variability in clinical outcomes across different samples worldwide. This is further corroborated by the difference between the characteristics of the current HR sample and other studies in this field. Although this is probably due to a variety of factors, including both biological and psychological components, certain profiles of substance use could potentially play a significant part in the evolution of these presentations. Therefore, substance use may well represent a clinical domain that requires further emphasis and more detailed consideration in future studies.

## Abbreviations

CAARMS: Comprehensive assessment of at-risk-mental-states
HR: Clinical high-risk for psychosis
HV: Healthy volunteers
MINI: Mini international neuropsychiatric interview
PANSS: The positive and negative syndrome scale
